# The Importance of Hormesis to Public Health

**DOI:** 10.1289/ehp.8606

**Published:** 2006-07-10

**Authors:** Ralph Cook, Edward J. Calabrese

**Affiliations:** 1 RRC Consulting, LLC, Midland, Michigan, USA; 2 School of Public Health and Health Sciences, Department of Environmental Health, University of Massachusetts, Amherst, Massachusetts, USA

**Keywords:** biphasic, dose response, hormesis, J-shaped, risk assessment, U-shaped

## Abstract

**Background:**

Hormesis is a specific type of nonmonotonic dose response whose occurrence has been documented across a broad range of biological models, diverse types of exposure, and a variety of outcomes. The effects that occur at various points along this curve can be interpreted as beneficial or detrimental, depending on the biological or ecologic context in which they occur.

**Objective:**

Because hormesis appears to be a relatively common phenomenon that has not yet been incorporated into regulatory practice, the objective of this commentary is to explore some of its more obvious public health and risk assessment implications, with particular reference to issues raised recently within this journal by other authors.

**Discussion:**

Hormesis appears to be more common than dose–response curves that are currently used in the risk assessment process [e.g., linear no-threshold (LNT)]. Although a number of mechanisms have been identified that explain many hormetic dose–response relationships, better understanding of this phenomenon will likely lead to different strategies not only for the prevention and treatment of disease but also for the promotion of improved public health as it relates to both specific and more holistic health outcomes.

**Conclusions:**

We believe that ignoring hormesis is poor policy because it ignores knowledge that could be used to improve public health.

The acceptance of the concept of hormesis, a specific type of nonmonotonic dose response, has accelerated in recent years (Academie Nationale de Medecine 2005; Cendergreen et al. 2005; Kaiser 2003; Puatanachokchai et al. 2005; Randic and Estrada 2005; Renner 2003). Nonetheless, it has not been without its detractors. One article critical of the concept was published last year in *Environmental Health Perspectives* (Thayer et al. 2005). It provided a summary of the major points of contention and thus a convenient vehicle for us to use in responding to opposing perspectives.

Although Thayer et al. (2005) tacitly acknowledged the existence of the phenomenon, they argued that no consideration should be given to hormesis in assessments of chemical risks for regulatory purposes. We disagree with their conclusion, but believe some of their points have merit—with important clarifications. We also believe that the proper understanding and utilization of hormesis will do a much better job of both protecting and promoting public health than the policy-based defaults that are currently in use.

Contrary to the assertion of Thayer et al. (2005) that hormesis is rare, it is a ubiquitous natural phenomenon (Calabrese and Blain 2005). Although given many names, hormesis has been observed in the fields of medicine (Brandes 2005; Celik et al. 2005), molecular biology (Randic and Estrada 2005), pharmacology (Chiueh et al. 2005), nutrition (Lindsay 2005), aging and geriatrics (Lamming et al. 2004; Rattan 2004a, 2004b, 2004c, 2005; Sinclair et al. 2005), agriculture (Brandt et al. 2004; Shama and Alderson 2005), microbiology (Brugmann and Firmani 2005), immunology (Dietert 2005; Liu 2003), toxicology (Stebbing 2000), exercise physiology (Radak et al. 2005), and carcinogenesis (Fukushima et al. 2005)—literally, across the biological spectrum. It has also been observed in relation to disparate outcomes from the isolated single cellular process to the more holistic (e.g., growth, longevity, disease, death) that likely result from a complex interplay of multiple factors and mechanisms (Calabrese 2005d).

In some fields, such as pharmacology and nutrition, these findings have been used directly or indirectly to improve human health. In others, they have been dismissed as artifacts and ignored (Calabrese 2005b). For example, certain micronutrients and vitamins can be toxic at high levels, even though low levels are essential to good health (Axelrod et al. 2004); even lower levels lead to deficiency conditions that are still problems of major public health significance in some parts of the world. Unfortunately, it is less well known that the phenomenon has also been documented for a host of other chemicals, including inorganic preservatives, antineoplastic drugs, pesticides, and various industrial chemicals (both individual agents and mixtures) (Calabrese 2005d).

Mechanistic research conducted on some of these agents explains the underlying biological actions related to the respective agents at both low and high exposures (Calabrese 2005a, 2005c; Calabrese and Baldwin 2001a; Levchenko et al. 2004; Szabadi 1977). The same cannot be said about many of the policy-based defaults that are routinely used in the current risk assessment process employed for the development of occupational and environmental health policy. Especially with regard to low-level exposures, both the hypothetical shape of the curves associated with these defaults and their presumptive underlying mechanisms are based on assumptions that are largely untested or untestable.

## Dose–Response Curve

**Figure 1:**
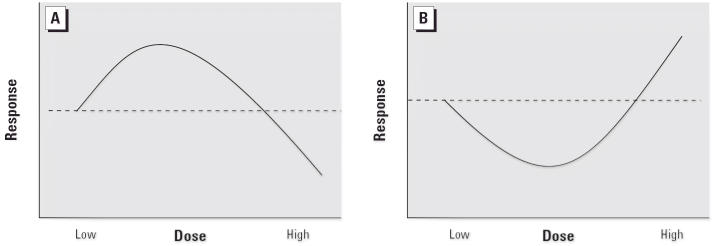
Schematic forms of the hormetic dose response. (*A*) The most common form of the hormetic dose–response curve showing low-dose stimulatory and high-dose inhibitory responses (β- or inverted U-shaped curve). (*B*) The hormetic dose–response curve depicting low-dose reduction and high-dose enhancement of adverse effects (J- or U-shaped curve).

The hormetic curve (Figure 1) can be most easily understood in terms of low-dose stimulation and high-dose inhibition. Depending on the outcome of interest, this interplay results in either a J-shaped or inverted J-shaped dose response (sometimes called “U-shaped” or “inverted U-shaped,” or “biphasic” or “β-curve”). The point at which the hormetic curve crosses the reference level of response (i.e., the threshold) is the zero equivalent point (ZEP).

**Figure 2:**
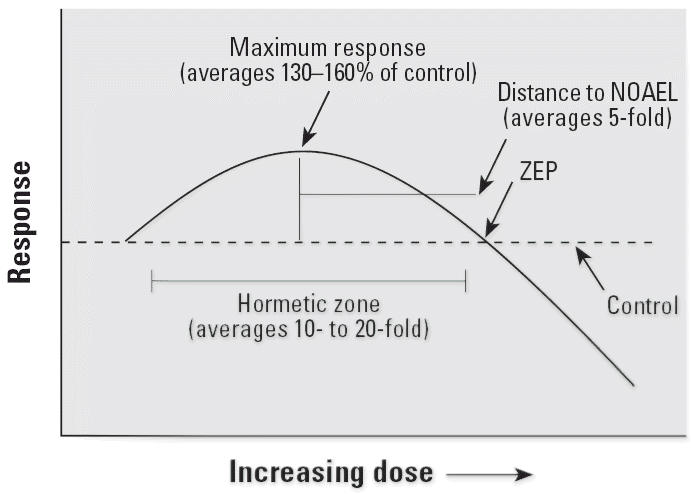
Dose–response curve showing the quantitative features of hormesis. NOAEL, no observed adverse effect level.

Thayer et al. (2005) believe the term hormesis would be “better described by the more general term ‘nonmonotonic’ dose responses.” This suggestion does not offer any advantages and, in fact, is simply too general. Hormesis is a specific type of nonmonotonic dose response, one with characteristic quantitative features (Figure 2) relating to the magnitude of the response, relationship of the point of maximum stimulation to the ZEP, the width of the stimulatory response, and temporal features (Calabrese and Baldwin 2001b). The term “nonmonotonic” is less precise and would simply lump unrelated phenomena together. Hormesis is a much more focused term and therefore preferable.

Although we agree with Thayer et al. (2005) that “there is a need to address nonmonotonic dose–response relationships in the risk assessment process,” our particular interest is in that subset classified as hormesis because of its ubiquity and, therefore, its potential importance to public health. In fact, extensive review of the literature has demonstrated that below-NOEL (no observed effect level) responses are overwhelmingly more consistent with hormesis than with its rival models, including linear no-threshold (LNT) dose response (Calabrese and Baldwin 2001b, 2003).

## Beneficial versus Harmful

Thayer et al. (2005) argued that stimulatory responses are not always beneficial and that some may be harmful. We agree. In fact, either inhibitory or stimulatory effects may be harmful or beneficial, a point that we have made on numerous occasions; one example was presented by Calabrese and Baldwin (2002a): “even though hormesis is considered an adaptive response, the issue of beneficial/harmful effects should not be part of the definition of hormesis, but reserved to a subsequent evaluation of the biological and ecological context of the response.” In the text, numerous examples were offered. For instance, in clinical medicine, whether a particular treatment is beneficial or not differs when viewed from the perspective of the patient or of an attacking organism. A dose that is sufficient to inhibit the organism likely will cure the patient; however, the patient may die as a result of a dose that is too low, because such a dose may stimulate the invading organism to the extent that it overwhelms the body’s natural defenses.

Even in situations in which deleterious impacts on humans might occur, either in the general population or in sensitive subgroups, it is important to recognize that *a*) if hormesis continues to be ignored by tradition or policy, those effects likely will be overlooked; *b*) a problem overlooked is a problem that can never be properly addressed; and *c*) whether there really is or is not a problem, especially one that potentially could occur indirectly, can be documented only by means of empirical data (data collected via observation and experiment on health effects and their underlying mechanisms).

Nonetheless, it is also important to recognize that striving to reduce some exposures ever lower, simply because it is possible, may not only be unnecessary for the protection of public health, but it may be counterproductive. In a state of ignorance, “erring on the side of caution” may not be cautionary; it may simply be an error—one that carries with it a host of social penalties and/or lost opportunities. This presumptive “precautionary” approach arguably had utility in the past, as pointed out by Johnson (2004) in a commentary on the U.S. Environmental Protection Agency (EPA) report *An Examination of EPA Risk Assessment Principles and Practices* (Risk Assessment Task Force 2004), but it is a philosophy that became prominent during the middle of the last century, when many of the technologies that are currently available simply did not exist. The time has come to move on, to begin making risk-based decisions founded more on actual biological data rather than on convenient statistical assumptions (Kathren 1996).

## Exposure Limits

As Thayer et al. (2005) noted, an environmental policy that mandates an optimal point level of exposure makes no sense, if for no other reason than it would be technically impossible to maintain. On the other hand, given a situation where the nadir of the J-shaped curve equated to benefit, neither does an exposure limit based on the LNT model because such a limit would have the net effect of diminishing or eliminating a benefit. With hormesis, any exposure limit below the ZEP would protect the general public against the risk of disease in excess of background—including the hypothetical 1 in a million inherent to the LNT approach—but an exposure limit in the range of the maximum stimulation could promote appreciable benefits in public health. Note the differentiation between “protect” and “promote.” The former is basically an attempt to maintain the frequency of disease near background; the latter relates to reducing the frequency of disease below background (i.e., improving the health of the general public). Any exposure limit established in a fairly broad range around the nadir of the hormetic curve would accomplish that goal to a greater or lesser extent. It logically follows that any exposure limit appreciably below the nadir could equate to a lost opportunity.

## All Induced Effects

Thayer et al. (2005) called for health decisions to be based on “all induced effects.” We agree, at least with all effects that likely result from levels of exposure that actually occur in the environment. The reliance on a sentinel outcome in the formulation of health policy, irrespective of whether the outcome is beneficial or detrimental, makes no sense, especially in situations where the agent clearly is associated with multiple outcomes.

Ethanol is a case in point. As Lin et al. (2005) reported, ingestion of alcohol is associated with nonlinear (hormetic) dose–response curves for death from all causes, death from cancer (presumptively all types), and death from cardiovascular disease among Japanese men. For all three disease categories, the moderate intake of 0.1–22.9 g/day alcohol (equivalent to one to two drinks per day) was associated with statistically significant decreases in the order of 20% relative to the reference (nondrinkers, relative risk = 1) and the highest level of consumption (≥69 g/day) was associated with statistically significant elevations of approximately 40%. Favorable mortality patterns, albeit not quite as dramatic, were also noted for Japanese women. Among men, the decrease in the risk for all-cause mortality was greater in never-smokers than in ever-smokers. However, Lin et al. (2005) also reported elevated risks for death from injuries and external causes at all levels of consumption (albeit only the highest dose was statistically significant).

None of the findings are particularly surprising, and one certainly should not drink and drive. However, while health care providers caution against its abuse, they are increasingly advising their patients of the protective advantages of the moderate, routine consumption of ethanol. They are doing this in spite of the fact that the mechanisms related to harm are much better understood than the mechanisms of benefit, especially for such a broad category such as death from all causes. In essence, the clinicians are making their decisions based on a simple risk–benefit calculation. In their study, Lin et al. (2005) reported approximately 175 fewer deaths from all causes and 7 excess deaths from injuries and external causes, a beneficial ratio of 25:1 for the group who consumed moderate daily amounts of alcoholic beverages.

## Mechanisms of Action

Thayer et al. (2005) contended that little is known about the mechanisms underlying hormesis. Further, they argued that, in the absence of comprehensive mechanistic foundations, hormetic-like dose–response relationships are meaningless. The first assertion is incorrect, and the second, shortsighted.

It is a myth that little is known about hormetic mechanisms. In fact, the case is just the opposite. As early as 2001, a series of articles was published on a range of endogenous agonists [prostaglandins (Calabrese 2001i), nitric oxide (Calabrese 2001g), estrogens and related compounds (Calabrese 2001e), androgens (Calabrese 2001c), adrenergic agonists (Calabrese 2001b), adenosine (Calabrese 2001a), 5-hydroxytryptamine (Calabrese 2001f), dopamine (Calabrese 2001d), and opiates (Calabrese 2001h)] that display hormetic biphasic dose responses. These articles documented that the mechanisms of biphasic dose responses were clearly established to the level of receptor and, in a number of cases, to further levels of molecular detail. Later assessments have identified dozens of hormetic mechanisms for immune responses (Calabrese 2005c) and for responses in tumor cell lines (Calabrese 2005a). At that time, more than two dozen receptor systems demonstrated hormetic dose responses. In general, the receptor systems display such biphasic dose responses when a single agonist has differential affinity for two opposing receptor subtypes, a concept that was first described in detail by Szabadi (1977). These molecular mechanism–oriented concepts and examples have been both reaffirmed and extended in recent work by Levchenko et al. (2004), who dealt with regulatory modules that generate biphasic dose–response relationships. As more research is conducted, it is likely that even more mechanisms will be discovered that operate at the level of the molecule, cell, tissue, or total organism.

As previously implied (Calabrese 2005a, 2005c; Thayer et al. 2005) additional research is needed to expand our understanding of hormesis; however, it is shortsighted to assume that comprehensive mechanistic knowledge is necessary before an effect has been (or can be) considered in health policy. The history of medicine and public health is replete with examples of new insights supplanting previously “well-established” concepts of disease and how they should be addressed; for example, asbestos, vaccinations, penicillin, and yellow fever. The more numerous, consistent, and coherent the findings of benefit or harm, the more readily they were accepted and acted upon even in the absence of comprehensive mechanistic explanations. To argue that hormetic mechanisms require a higher level of understanding is simply an example of a double standard designed to accomplish little more than maintain the status quo.

Science is an iterative process of theory, test, confirmation, and refinement to fit new data and ideas. If a concept cannot be replicated or sufficient explanatory data developed, it will be rejected, as was the theory of cold fusion. Alternatively, if new observations of benefit or harm can be replicated, the public is best served by acting upon them.

By way of example, until the latter part of the 20th century, upper gastrointestinal inflammation and ulcers were thought to be caused by excessive stomach acids. Interventions, some quite invasive and dangerous, were designed to block the production or actions of gastric juices. In the 1980s, two Australian investigators reported that, in most cases, these problems had an infectious etiology (Gupta 2005). Initially, the medical community had great difficulty accepting these findings, in part because they rendered so much previous work and opinion obsolete. It is now acknowledged that an infectious agent, *Helicobacter pylori*, is the major causative agent for approximately 90% of gastric ulcers and 75% of duodenal ulcers (and quite possibly certain gastric malignancies). Although the ultimate mechanisms by which these occur are not known, many of the problems currently are treated successfully with antibiotics (Gupta 2005).

There is one final problem with relying too heavily on mechanistic research before acting on evidence of benefit or harm. As noted in a previously published article (Calabrese 2005a),

Problematic in the general area of research is that investigators who report findings on in vitro tumor cell proliferation do not typically cite responses in other systems such as the immune that could affect tumor responses, thereby rarely approaching an integrative assessment of the whole organism.

This suggests that such *in vitro* work—in isolation—cannot be used to make the risk–benefit calculations like those that we described above for alcohol. Mechanistic research, while certainly valuable, plays a much more important role in the development of strategies for prevention or intervention.

## High Risk Groups

In the recent government report *An Examination of EPA Risk Assessment Principles and Practices*, the Risk Assessment Task Force (2004) pointed out that it is not agency policy to protect the most sensitive in the general population, just the more sensitive. With proper knowledge, we think it may be possible to protect both subgroups against excess risk and still promote decreased risk among those in the general population with “normal” sensitivity.

Responding to concerns expressed by Lave (2001), Calabrese and Baldwin (2002b) pointed out that previous work had never addressed this critical area in the risk assessment process. They used the hormesis database to explore the responses of potential high-risk individuals and highly sensitive species to toxic substances. This analysis indicated that those at increased risk typically displayed the hormetic response; it just shifted to the left on the dose–response spectrum. In setting exposure limits for a population that included such a subset of individuals, any limit set below the ZEP for the sensitive individuals would protect both sensitive and normal individuals against excess disease over background. That limit likely also could provide some additional benefits to the normal individuals (i.e., decrease the risk to that group and thus promote improved public health).

Calabrese and Baldwin (2002b) also found that, in about 20% of the cases, a hormetic response was not seen and may have been a factor in the observed increased risk. Protecting this group is a challenge, no matter what the underlying biological model. Calabrese and Baldwin (2002b) concluded that there is no conceptual or technical conflict unique to hormesis and high-risk groups. This concept is simply another component to an overall sophisticated analysis of a population-based dose response.

We fully agree that an agency could make the decision to lower the exposure limit below the range that optimized health for the general public, for example, to protect the unborn or some other segment of the population that had been shown to be more sensitive to the putative agent. In fact, this decision might even be made to protect a susceptible plant or animal species; but all of these decisions, in the vernacular of the U.S. EPA (Risk Assessment Task Force 2004), would have to be “transparent.” In other words, it would have to be acknowledged that the general public likely could suffer an increased risk to a preventable burden of disease as a result of such a decision.

## Multiple Chemical Exposures

Thayer et al. (2005) emphasized the need to consider all chemical exposures in any risk assessment process. As is the case of high-risk groups, this is not any more of a technical issue for hormesis than it is for any other dose–response model. Mixture data are generally limited, but there are sufficient data on mixtures to indicate that hormetic effects would routinely occur. Hormetic effects have been reported for complex mixtures such as well-characterized wastewater effluent (Walsh et al. 1980) and petroleum mixtures (Laughlin et al. 1981). They have also been reported for more simplified limited chemical mixtures (Flood et al. 1985; Gennings et al. 2002).

## FDA Regulation of Hormesis

Thayer et al. (2005) maintained that any beneficial effects (but apparently not concurrent detrimental effects) related to environmental exposures need to be under the regulatory control of the Food and Drug Administration (FDA). In part, they suggest that is because the proponents of hormesis want “increased environmental exposures to toxic and carcinogenic agents.” That is a misrepresentation of our position. What we are advocating, with the few exceptions noted above, is that environmental exposures only need to be lowered to the range that maximizes public health, because driving them much lower would place the public at unnecessary risk to preventable disease or death. Therefore, a regulation that mandates limits appreciably below the nadir of the hormetic curve would be bad public health policy and should require justification, with supporting data, from the agency proposing the policy. The FDA would not be involved with this process.

## Radiation Hormesis

Thayer et al. (2005) provided a quotation from the 2005 Biological Effects of Ionizing Radiation (BEIR) VII report [National Research Council (NRC) 2005] which they implied supported their contention that hormesis should be ignored:

The assumption that any stimulatory hormetic effects from low doses of ionizing radiation will have a significant health benefit to humans that exceeds potential detrimental effects from the radiation exposure is unwarranted.

For a number of reasons, that reference was selective and misleading. First, the quotation was incomplete. The sentence did not end with the word “unwarranted”; it actually ended with “unwarranted at this time.” Second, Thayer et al. (2005) did not mention that among the 12 research needs recommended by the BEIR VII committee, two involved hormesis (NRC 2005). Third, Thayer et al. did not reference the report from the Academie Nationale de Medecine (2005).

Both the BEIR committee (NRC 2005) and the French committee (Academie Nationale de Medecine 2005) issued their reports concerning the health effects of ionizing radiation at approximately the same time; therefore, both presumptively had access to the same literature. They both recommended research on hormesis, but the Academie Nationale de Medecine (2005) went further in that they challenged the validity of the LNT model and stated that “the importance of hormesis should not be overlooked.”

## Conclusions

Hormetic dose–response curves have been observed for a large number of individual agents and various mixtures, across the biological spectrum, and for responses ranging from the cellular level to broad categories of disease (Calabrese and Baldwin 2001c; Calabrese and Blain 2005). They are too numerous to be dismissed as artifacts and too important to be ignored.

Much in this field has changed over the last few years. The topic has been included in leading toxicologic and risk assessment texts, taught at graduate level courses in toxicology, and discussed at major professional meetings. Furthermore, a growing number of international governmental advisory bodies have begun to give detailed consideration to the concept and its risk assessment implications, and how these may be incorporated into the regulatory process.

A great strength of the hormetic model not addressed by Thayer et al. (2005) is that it has the capacity to be tested and thereby validated or rejected with experimental data in the observable zone. This is in contrast to the linear-at-low-dose model that U.S. government agencies currently use to estimate cancer risk.

The hormetic model also provides decision makers in regulatory agencies with a much broader array of options in the risk assessment process; with the hormetic model, they can consider potential benefits, as well as risks, to health among the general public and specific subgroups. Therefore, it will allow decision makers to consider not only how to protect health but, more importantly, how to optimize it. Admittedly, these choices, while attractive, will also be challenging, in part because they may be more complex and, in part, because they may tend to bring various subgroups in the population together to debate one group’s health benefit against another group’s health risk. This will make the stakeholder concept much more dynamic and involve a broader array of subgroups in the population.

The time has come to move away from the LNT model, certainly move away from it as the default. Acceptance of the reality of hormesis by various government agencies in the United States will likely accelerate the acquisition of knowledge about this phenomenon. More resources will become available to conduct experiments specifically designed with hormesis in mind. More reasoned discussions will take place among risk assessors and risk managers. We believe that all of these will set the stage for actions that, directly and indirectly, will result in substantial improvements in the health of both the general public and the environment.
